# Decoding the long-term safety of anti-CGRP (receptor) mAbs: a meta-analysis and systematic review

**DOI:** 10.1186/s10194-025-02256-0

**Published:** 2026-01-03

**Authors:** Carolin Luisa Hoehne, Lucas Hendrik Overeem, Margarita Sanchez-Del-Rio, Christiana Deligianni, Raquel Gil-Gouveia, Jan Versijpt, Faisal Mohammad Amin, Christian Lampl, Kristina Ryliskiene, Erling Tronvik, Gianluca Coppola, Philip R. Holland, Antoinette MaassenVanDenBrink, Paolo Martelletti, Uwe Reuter

**Affiliations:** 1 Department of Neurology, Charité Universitätsmedizin Berlin, Corporate member of Humboldt University and Frei Universität Berlin, Charitéplatz 1, Bonhoefferweg 3, 10117 Berlin, Germany; 2 Department of Neurology, Clínica Universidad de Navarra, Calle Marquesado de Santa Marta, 1, Madrid, 28027 Spain; 3 First Department of Neurology, Aeginition Hospital, Medical School National and Kapodistrian University of Athens, Athens, Greece; 4 Neurology Department, Athens Naval Hospital, Athens, Greece; 5 Neurology Department, Hospital da Luz, Lisboa, Portugal; 6 Center for Interdisciplinary Research in Health, Universidade Católica Portuguesa, Lisboa, Portugal; 7 Department of Neurology, Universitair Ziekenhuis Brussel, Brussels, Belgium; 8 Neuroprotection and Neuromodulation Research Group, Center for Neurosciences, Vrije Universiteit Brussel, Brussels, Belgium; 9 Department of Neurology, Danish Headache Center, Copenhagen University Hospital - Rigshospitalet, Copenhagen, Denmark; 10 Department of Neurology, Krankenhaus Barmherzige Brüder, Linz, Austria; 11 Neurology and Neurosurgery Clinic, Institute of Clinical Medicine, Faculty of Medicine, Vilnius University, Vilnius, Lithuania; 12 NorHead, Norwegian Headache Research Centre, NTNU, Norwegian University of Science and Technology, Trondheim, Norway; 13 Department of Neuromedicine and Movement Science, Norwegian University of Science and Technology, Trondheim, Norway; 14 Department of Medico-Surgical Sciences and Biotechnologies, Sapienza University of Rome Polo Pontino ICOT, Latina, Italy; 15 Headache Group, Wolfson Sensory Pain and Regeneration Centre, Institute of Psychiatry, Psychology, and Neuroscience, King’s College London, London, UK; 16 Division of Pharmacology and Vascular Medicine, Department of Internal Medicine, Erasmus MC University Medical Center Rotterdam, PO Box 2040, Rotterdam, CA 3000 The Netherlands; 17 School of Health, Unitelma Sapienza University of Rome, 00161 Rome, Italy

**Keywords:** Migraine, Galcanezumab, Fremanezumab, Erenumab, Eptinezumab, Long-term safety, CGRP

## Abstract

**Objective:**

To evaluate the long-term safety (≥12 months) of monoclonal antibodies (mAbs) targeting the calcitonin gene-related peptide (CGRP) or its receptor in migraine prevention by synthesising evidence from clinical trials and real-world studies. We focus on drug discontinuation due to adverse events and the type and frequency of adverse events. This is the first review to analyse the effects of the long-term use of all anti-CGRP (receptor) mAbs, aiming to provide novel insights for clinical practice and future treatment strategies.

**Methods:**

We systematically searched PubMed, Cochrane Library, and ClinicalTrials.gov for studies with ≥12 months of anti-CGRP (receptor) mAb use between January 2013 and April 2025. A random-effects meta-analysis of proportions (logit transformation, inverse variance weighting, restricted maximum likelihood) was performed to estimate pooled discontinuation and adverse event rates. Risk of bias was assessed using the ROBINS-I score.

**Results:**

From a total of 1,499 records, 14 met the inclusion criteria and were eligible for data analysis. These 14 records corresponded to 11 individual studies with observational durations all exceeding 12 months. Seven studies investigated erenumab, two eptinezumab, and one each fremanezumab and galcanezumab. All studies were judged to have a severe risk of bias due to their underlying design. The overall pooled proportion of treatment discontinuation for any reason among patients receiving anti-CGRP (receptor) mAbs was 23%, whereas the pooled proportion of discontinuation specifically due to adverse events was substantially lower at 3%. Time-trend analysis showed that adverse event–related discontinuation remained low (<5%) beyond the first year, while overall adverse event incidence was high at baseline (>70%) but did not further increase with prolonged follow-up.

**Conclusion:**

Evidence on long-term use of anti-CGRP (receptor) mAbs over 12 months remains limited, but our analysis indicates good tolerability with consistently low adverse event-related discontinuation, no emergent safety signals, and largely non-serious, stable adverse event profiles. However, heterogeneity and study-level bias warrant cautious interpretation, highlighting the need for long-term clinical studies and continued real-world surveillance (e.g. registries).

**Clinical trial number:**

Not applicable.

**Supplementary Information:**

The online version contains supplementary material available at 10.1186/s10194-025-02256-0.

## Introduction

Migraine is a chronic, disabling neurological disorder affecting over one billion people globally, with a higher prevalence in women and a peak incidence between the ages of 25 and 55 [1, 2]. Given the high disease burden, there is a clear need for preventive therapy in approximately 40% of affected individuals. Conventional oral prophylactic treatments have historically been associated with limited efficacy, poor tolerability and subsequent high discontinuation rates [3]. In recent years, calcitonin gene-related peptide (CGRP) has emerged as a key target in migraine pathophysiology, leading to the development of monoclonal antibodies (mAbs) for migraine prevention that target either CGRP to prevent its interaction with the receptor (e.g., galcanezumab, fremanezumab, eptinezumab) or its receptor (erenumab) [4]. Several years later, small-molecule CGRP receptor antagonists (gepants) such as ubrogepant, rimegepant, and atogepant were developed for both acute and preventive migraine treatment [5].

The first anti-CGRP receptor mAb, erenumab, was launched in 2018, and this substance class has transformed the landscape of migraine prevention ever since [6]. The clinical efficacy and tolerability of anti-CGRP (receptor) mAbs have been demonstrated in multiple large-scale, randomised controlled trials (RCTs) and real-world observational studies for both episodic (EM) and chronic migraine (CM), with many studies showing significant reductions in monthly migraine days, acute medication use and improved quality of life. These agents are now recommended in several national and international guidelines, often as a first-line preventive option for migraine [3, 7].

Long-term safety is of particular importance for preventive migraine therapies, especially given the chronic nature of the disorder and the likelihood that patients may remain on treatment for several years. Anti-CGRP (receptor) mAbs are characterized by longer plasma half-lives (approximately 28 days) compared with traditional oral prophylactics, resulting in sustained systemic exposure. However, the robustness of long-term safety evidence remains variable. Data from registries and pragmatic cohort studies extending beyond 12 months are still emerging. Furthermore, there is limited comparative insight into how safety outcomes observed in real-world data align with those reported in RCTs and long-term extension studies.

Given these considerations, we performed a systematic review and meta-analysis to evaluate the long-term safety profile of anti-CGRP (receptor) mAbs, including evidence from both clinical trials and real-world studies. Specifically, our goals were to determine whether sufficient data support the long-term safety of anti-CGRP (receptor) mAbs beyond 12 months of use and to identify and measure the type and frequency of adverse events (AEs) associated with long-term (≥12 months) exposure.

By synthesising available data from diverse study designs and patient populations, this review aims to provide a comprehensive evaluation of the long-term safety of anti-CGRP (receptor) mAbs, with implications for clinical decision-making and potentially guideline development.

## Methods

This review was carried out following the guidelines outlined by the Preferred Reporting Items for Systematic Reviews and Meta-Analyses (PRISMA). As the analysis is based solely on data from existing published literature and does not involve the collection of new data from human or animal participants, no ethical approval or informed consent was required.

### Objective and search strategy

The primary objective of this systematic review and meta-analysis was to assess the long-term safety profile of anti-CGRP (receptor) mAbs for the prevention of migraine. Specifically, we aimed to determine whether sufficient safety data exist for treatment durations exceeding 12 months, to assess the discontinuation rate due to AEs as a specific safety outcome, and to summarise the type and frequency of AEs reported during long-term use.

To address these questions, we systematically reviewed studies that reported safety outcomes for erenumab, galcanezumab, fremanezumab, or eptinezumab in patients with episodic or chronic migraine, with a minimum duration of 12 months. We included all relevant studies published from January 2013 through April 2025. A systematic search of PubMed, the Cochrane Library, and ClinicalTrials.gov was carried out on April 4, 2025. Duplicate records were removed using EndNote 2025.

The search terms for PubMed were the following:

(galcanezumab[Title/Abstract]) OR (LY2951742[Title/Abstract])

OR (eptinezumab[Title/Abstract] OR (ALD403[Title/Abstract])

OR (fremanezumab[Title/Abstract]) OR (LBR101[Title/Abstract])

OR (erenumab[Title/Abstract]) OR (AMG334[Title/Abstract])

AND “long-term”[Title/Abstract] OR “12month”[Title/Abstract] OR “year*” [Title/Abstract]

AND “migraine”[Title/Abstract].

The Cochrane Library was searched for ‘galcanezumab OR eptinezumab OR fremanezumab OR erenumab OR CGRP-Antibody OR CGRP-Antibodies OR LY2951742 OR ALD403 OR AMG334 OR LBR101’ in Title, Abstract, Keywords; AND ‘migraine’ in Title, Abstract, Keywords; AND 12 months OR 1 year OR long term in Title, Abstract, Keywords.

ClinicalTrials.gov was included to ensure a comprehensive search and to identify completed long-term studies that might not yet have been indexed in bibliographic databases, as well as to verify the publication status of relevant trials. It was searched for completed studies (with results) involving anti-CGRP (receptor) mAbs (galcanezumab OR eptinezumab OR fremanezumab OR erenumab OR CGRP-Antibody OR CGRP-Antibodies OR LY2951742 OR ALD403 OR AMG334 OR LBR101) for migraine treatment conducted between January 1, 2013, and April 4, 2025.

Three reviewers (CLH, LHO and UR) independently screened the titles and abstracts identified in the initial search to determine their eligibility according to the inclusion criteria. Any discrepancies were resolved through consensus discussion. Subsequently, all authors evaluated the full-text articles.

#### Inclusion criteria

Studies were included if they: (1) reported safety and tolerability outcomes of anti-CGRP (receptor) mAbs with a treatment duration of at least 12 months or longer; (2) were designed as RCTs, open-label extension studies, or prospective or retrospective observational studies; (3) were published between 2013 and 2025.

#### Exclusion criteria

We excluded studies that: (1) were not written in English; (2) did not contain original data, such as reviews or meta-analyses, or were not full-text publications, including letters, conference posters, or conference abstracts; (3) reported data from preclinical, animal, or mechanistic studies; (4) did not report safety outcomes or adverse events, or presented data that could not be extracted or converted for quantitative synthesis; (5) analysed only subgroups of already included studies; (6) presented pooled data of already included studies.

### Data extraction and analysis

Data were extracted on study design, duration, population characteristics (sex, age, migraine subtype), the anti-CGRP (receptor) mAb used, dose, reported AEs and AE-specific discontinuation rates.

For each study, we first extracted the total number of reported AEs. When this number was not available, we used the number of treatment-emergent adverse events (TEAEs) as the best available estimate. Pregnancy was variably reported across studies, either as an AE or as a separate outcome. To ensure consistency and comparability, pregnancy was excluded from the analysed AE outcomes. Each study reported AEs according to its own incidence threshold (e.g., events occurring in >5% of patients or ≥4 events per 100 patient-years), and these thresholds were retained as reported without standardization (Supplementary Table 1). Finally, we grouped all reported AEs categories based on clinical relevance or organ system (Supplementary Table 2).

Several records referred to the same underlying study but reported outcomes at different timepoints and/or as interim analyses. To ensure consistency in the pooled meta-analysis of treatment discontinuation, only one record per study was included, specifically, the version reporting the latest available cumulative discontinuation data. As a result, not all eligible records were included in the pooled analysis.

Where discontinuation was reported separately for mutually exclusive subgroups (e.g., EM and CM), subgroup data were summed to obtain the total number of discontinuations per study or timepoint.

For the time trend analysis, all available time points across all records were incorporated. In cases where the same timepoint of one study was reported in multiple records, results were checked for consistency, and duplicate entries were excluded to avoid double-counting.

We performed a random-effects meta-analysis of proportions using the logit transformation to stabilize variance and normalize sampling distributions. Pooled estimates with 95% confidence intervals (CIs) were calculated for overall discontinuation, AE-related discontinuation, and for each AE category. Between-study heterogeneity was assessed using Cochran’s Q test, τ^2^, and the I^2^ statistic. Subgroup analyses were performed by antibody type and study design.

### Assessment for risk of bias

Risk of bias in non-randomised studies was evaluated using the ROBINS-I tool (V2, November 2024), which assesses bias across seven domains, including confounding, participant selection, classification of interventions, deviations from intended interventions, missing data, measurement of outcomes, and selection of reported results [8]. Each domain in the ROBINS-I tool was assessed to determine the overall risk of bias. Based on these assessments, studies were categorized as having low, moderate, or high risk of bias.

### Publication bias

We assessed publication bias visually using funnel plots and statistically using Egger’s regression test. We applied the trim-and-fill method to estimate the potential impact of unpublished studies.

## Results

### Study selection and overview

**Fig. 1:**
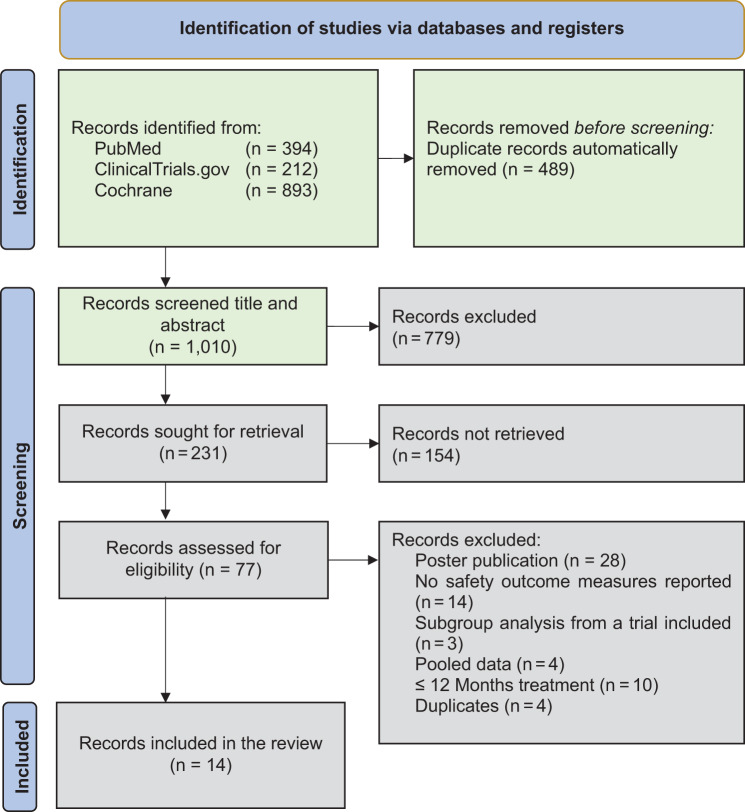
PRISMA flow diagram of study selection

The preliminary literature search identified 394 records from PubMed, 212 from ClinicalTrials.gov, and 893 from the Cochrane Library, published from January 2013 to April 2025. After removing duplicates in EndNote 2025, 1,010 records remained and were screened by title and abstract. Of these, 231 records were selected for full-text review, resulting in 77 records, of which 14 met the inclusion criteria and were eligible for data analysis (Fig. 1).

These fourteen selected records corresponded to eleven individual studies with varying observational durations. The majority of studies investigated the use of erenumab (*n* = 7), followed by two studies on eptinezumab, and one study each on galcanezumab and fremanezumab (Table 1). Of these eleven studies, seven were open-label extension trials following after a short-term placebo-controlled trial, one was a phase-III open-label study (participants in open-label *n* = 3,636), and three were real-world observational studies (participants in real-world *n* = 743). The study populations included patients with episodic migraine (EM), chronic migraine (CM), or both. Three studies specifically enrolled patients with a history of treatment failure: one study focused on CM patients who had failed at least three preventive therapies, and two studies included EM patients who had failed 2–4 prior preventive treatments. In total, the studies included 4,379 participants across a combined observational period of 1,052 weeks, ranging from a minimum of 60 to a maximum of 256 weeks.

**Table 1:** Overview of selected studies. NCT: national clinical trial, DBTP: double-blind treatment phase; EM: episodic migraine; CM: chronic migraine; na – not available

Author (Year)	NCT number (Study Title)	Time of Investigation	Area (Number of Centers)	Study Design	Duration in weeks (preceding DBTP)	Follow-Up Time	Dose	Study population	Number of participants	Female Sex N (%)	Age, years Mean (SD)
**Erenumab**											
Göbel et al (2024) [9]	NCT04084314 (APOLLON)	September 2019 to March 2023	Germany (79)	Open-Label- Extension	128 (24)	4 weeks	70 mg, 140 mg	EM, CM	701	608 (86.7%)	41.8 ± 12.3
Reuter et al (2024) [10]	NCT03096834 (LIBERTY)	March 2017 to January 2021	Europe and Australia (59)	Open-Label- Extension	144 (12)	6 months post-trial access phase	140 mg	EM for whom 2–4 prior preventatives had failed	240	196 (81.7%)	44.5 ± 10.5
Ferrari et al (2022) [11]	NCT03096834 (LIBERTY)	March 2017 to January 2021	Europe and Australia (59)	Open-Label- Extension	48 and 96 (12)	n/a	140 mg	EM for whom 2–4 prior preventatives had failed	240	196 (81.7%)	44.5 ± 10.5
Sakai et al (2021) [12]	NCT02630459	June 2016 to June 2019	Japan (43)	Open-Label- Extension	76 (24)	12 weeks	70 mg, 140 mg	EM	459	385 (83.9%)	43.9 ± 8.5
Ashina et al (2021) [13]	NCT01952574	August 2013 to November 2019	North America and Europe (59)	Open-Label- Extension	256 (12)	8–12 weeks	Initially 70 mg, then all 140 mg	EM	383	303 (79.1%)	41.3 ± 10.9
Goadsby et al (2021) [14]	NCT03096834 (LIBERTY)	March 2017 to January 2021	Europe and Australia (59)	Open-Label- Extension	52 (12)	n/a	140 mg	EM for whom 2–4 prior preventatives had failed	240	196 (81.7%)	44.5 ± 10.5
Tepper et al (2020) [15]	NCT02174861	June 2014 to May 2017	North America and Europe (69)	Open-Label- Extension	52 (12)	12 weeks	70 mg, 140 mg	CM	549	509 (92.7%)	42.5 ± 11.3
Ashina et al (2019) [16]	NCT01952574	August 2013 to November 2019	USA and Europe (59)	Open-Label- Extension	144 (12)	n/a	Initially 70 mg, then all 140 mg	EM	383	303 (79.1%)	41.3 ± 10.9
Gaul et al (2024) [17]	-	June 2019 and January 2021	Germany (105)	Real-World	48 or 96	n/a	70 mg, 140 mg	EM, CM	556	495 (89.0%)	45.0 ± 12.3
	(SPECTRE)										
Andreou et al (2022) [18]	-	na	United Kingdom (1)	Real-World	96	n/a	70 mg	CM for whom at least three preventives had failed	160	132 (82.5%)	48.0 ± 14.3
**Fremanezumab**											
Yoshida et al (2025) [19]	-	November 2021 to June 2022	Japan (1)	Real-World	96	n/a	675 mg	EM, CM	27	23 (85.2%)	41.7 ± 15.0
**Galcanezumab**											
Hirata et al (2021) [20]	NCT02959190	March 2017 to August 2019	Japan (44)	Open-Label- Extension	48 (24)	4 months	EM 120 mg	EM, CM	311	209 (67.2%)	44.7 ± 10.0
							EM 240 mg				
							CM 120 mg				
							CM 240 mg				
**Eptinezumab**											
Kudrow et al (2023) [21]	NCT02985398 (PREVAIL)	December 2016, to March 2019.	USA (20)	Open-Label- Phase 3	96	20 weeks	300 mg	CM	128	109 (85.2%)	41.5 ± 11.3
Ashina et al (2021) [22]	NCT04418765 (DELIVER)	June 2020 to September 2022	USA, Europe (96)	Open-Label-, Dose-Blinded Extension	48 (24)	n/a	100 mg	EM for whom 2–4 prior preventatives had failed	865	769 (88.9%)	43.9 ± 10.5
							300 mg				

### Assessing risk of bias with ROBINS-I

**Fig. 2:**
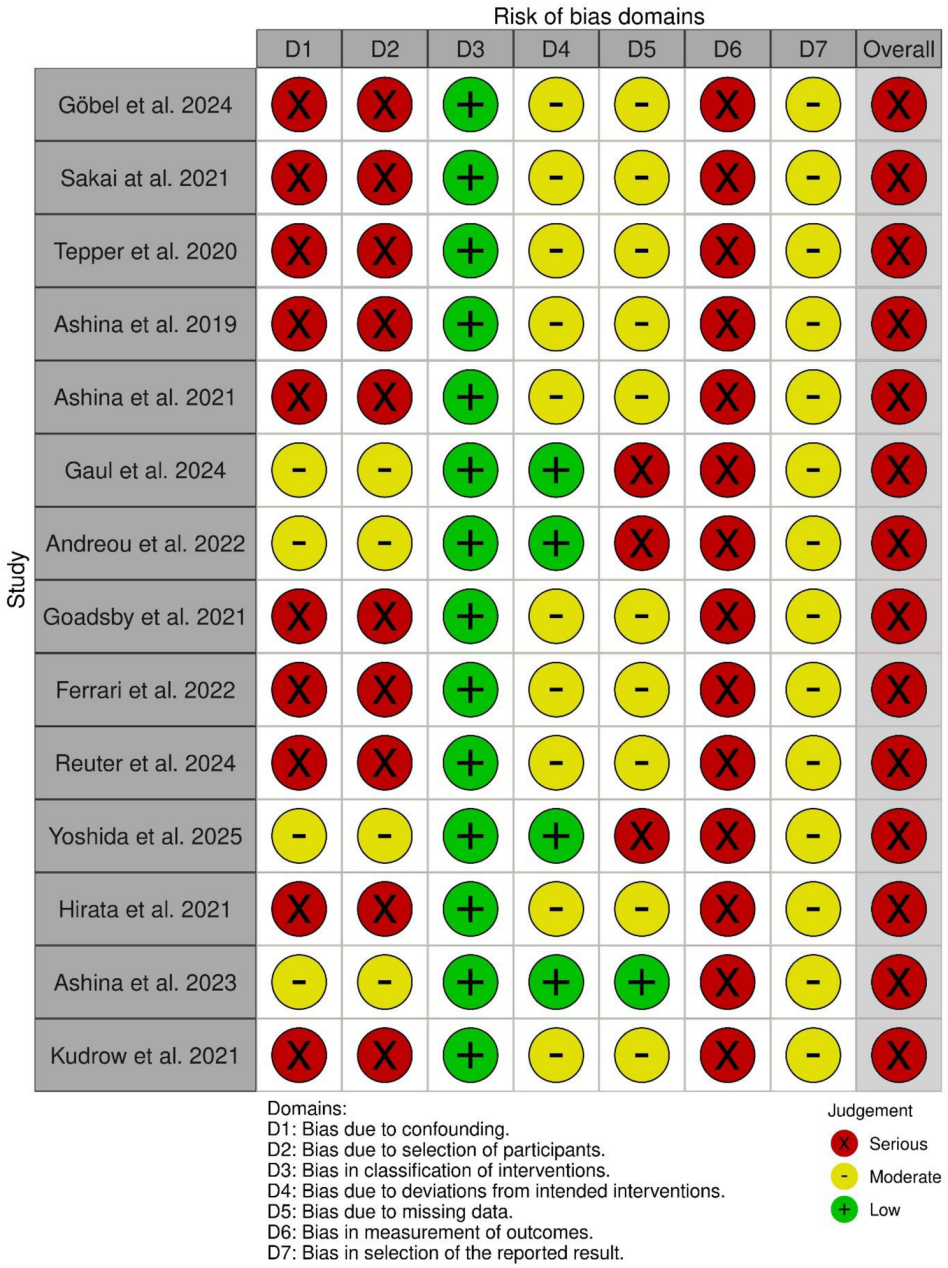
Risk of bias in non-randomised studies. Risk of bias assessment for included studies, conducted across seven domains using the ROBINS-I framework. Green = low risk, yellow = moderate risk, red = serious risk. Visualization was created with the *robvis* tool [23]

All included studies were judged at serious overall risk of bias, although the underlying reasons differed between the designs (Fig. 2). In open-label extension studies, the main concerns were the selection of participants, since only trial completers or responders were eligible to continue, and unblinded outcome measurement, as all key outcomes were patient-reported and could be influenced by treatment awareness. In contrast, real-world studies were most affected by confounding, due to non-random treatment and baseline differences. Across both types of studies, the classification of interventions was consistently low risk, while deviations from intended interventions, missing data, and selective reporting were generally rated at moderate risk.

### Treatment discontinuation in long-term CGRP monoclonal antibody studies

**Fig. 3:**
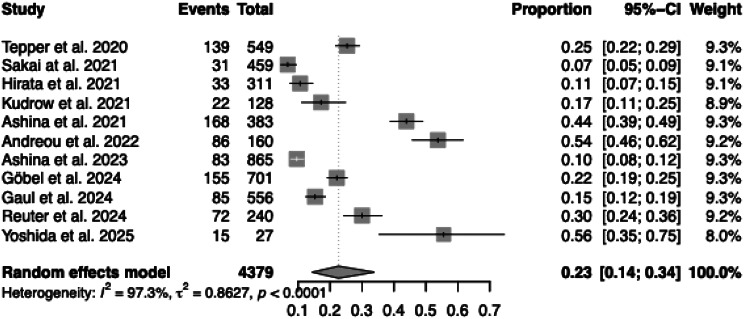
Total treatment discontinuation across studies. Forest plot showing the proportion of patients (events/total) who discontinued anti-CGRP (receptor) mAb treatment for any reason

The overall pooled proportion of treatment discontinuation for all reasons among patients receiving anti-CGRP (receptor) mAbs was 23% (95% CI: 14% to 34%, *p* < 0.0001; Fig. 3). Heterogeneity between studies was considerable (I^2^ = 97.3%, τ^2^ = 0.8627). Individual study estimates varied widely, ranging from 7% [12] to 56% [19].

**Fig. 4:**
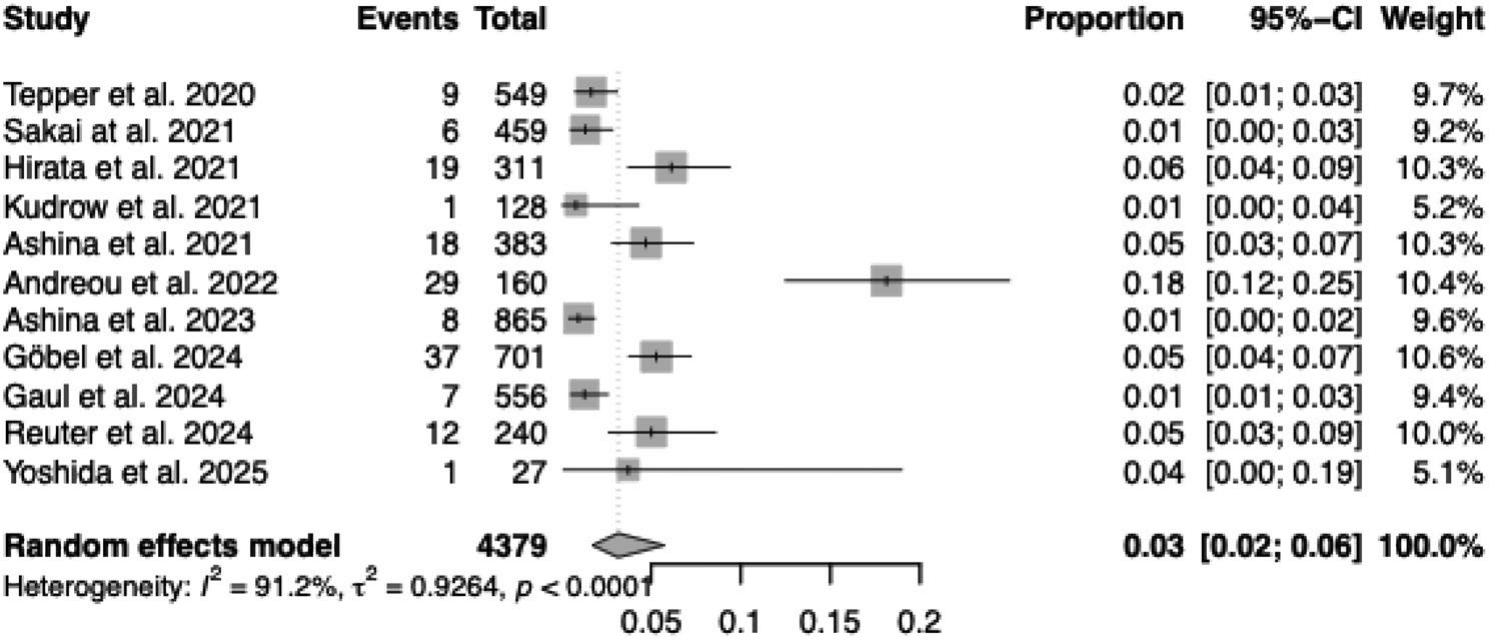
Discontinuation due to adverse events across studies. Forest plot showing the proportion of patients (events/total) who discontinued treatment due to adverse events (AEs)

In contrast, the pooled proportion of patients who discontinued treatment specifically due to AEs was significantly lower, at 3% (95% CI: 2% to 6%, *p* < 0.0001; Fig. 4). Although heterogeneity remained high (I^2^ = 91.2%, τ^2^ = 0.9264), most studies reported AE-related discontinuation rates below 6%. Only one study [18] reported a higher rate (18%).

Subgroup analysis by anti-CGRP (receptor) mAb revealed significantly different AE-related discontinuation rates (*p* < 0.001). Pooled estimates were highest for galcanezumab (6%), fremanezumab (4%), and erenumab (4%), and lowest for eptinezumab (1%). Only the results from erenumab were based on several studies, while the other anti-CGRP (receptor) mAbs were presented by smaller and fewer studies (Supplementary Figure 1).

An additional subgroup analysis comparing clinical open-label extension trials with real-world observational studies did not show a significant difference in AE-related discontinuation rates (*p* = 0.56), though real-world studies were fewer and more heterogeneous (Supplementary Figure 1).

In addition to AE-related discontinuation, several studies reported further reasons for treatment discontinuation. Across studies, the most frequent reasons were lack of efficacy and patient decision, with smaller numbers discontinuing due to new therapy initiation, loss to follow-up, or other study-specific reasons (Supplementary Table 3).

### Time trends

**Fig. 5:**
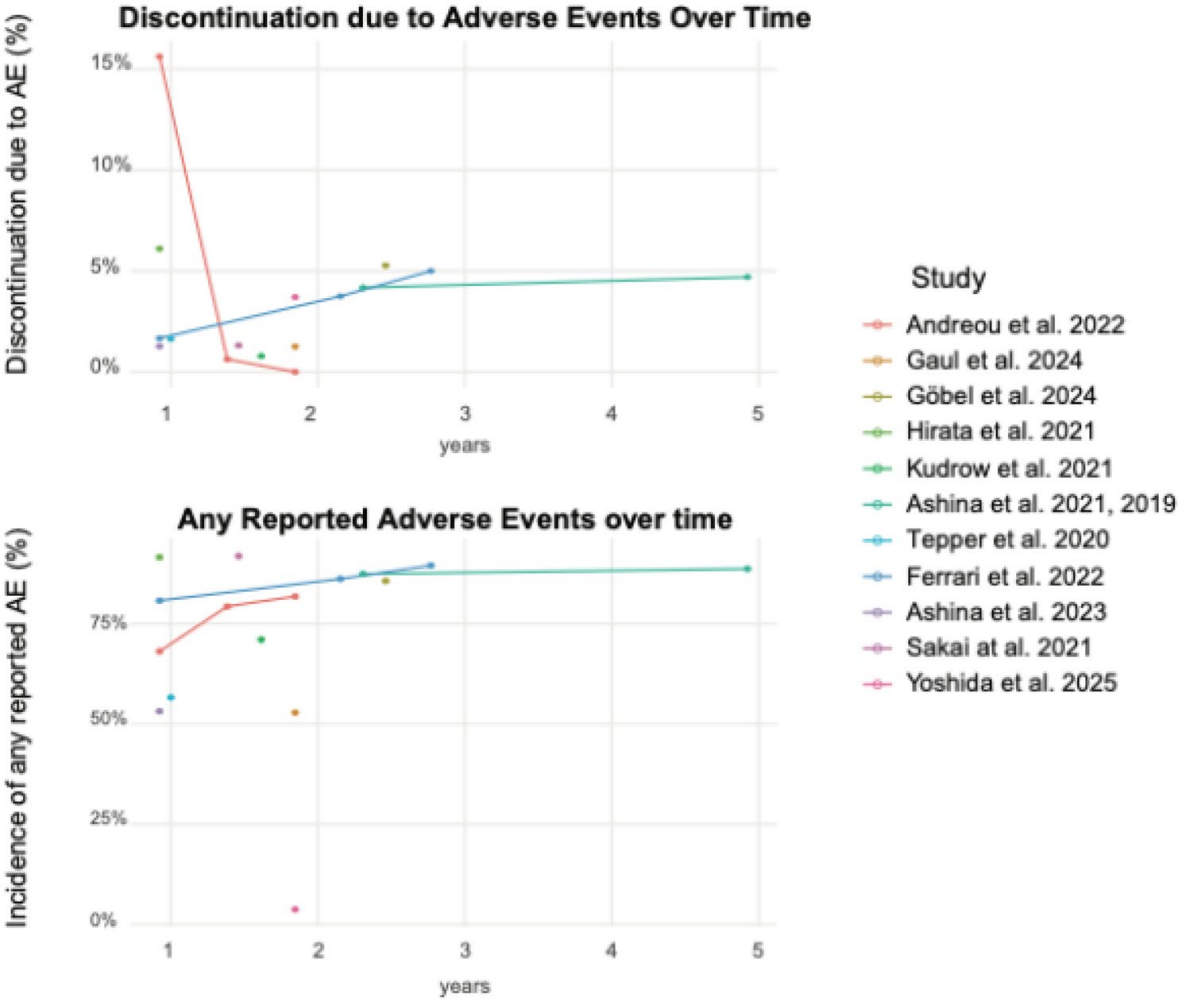
Time course in adverse event-related discontinuation and number of reported adverse events. AE: Adverse Events

AE-related discontinuation rates remained low throughout long-term follow-up, with most studies reporting proportions below 5% after the first year. One real-world study reported higher early discontinuation rates, which subsequently plateaued [18]. The incidence of any reported AE often exceeded 70% at the earliest timepoints and showed minimal increase with longer observation and only minor accumulation over extended follow-up (Fig. 5).

### Reported adverse events

**Fig. 6:**
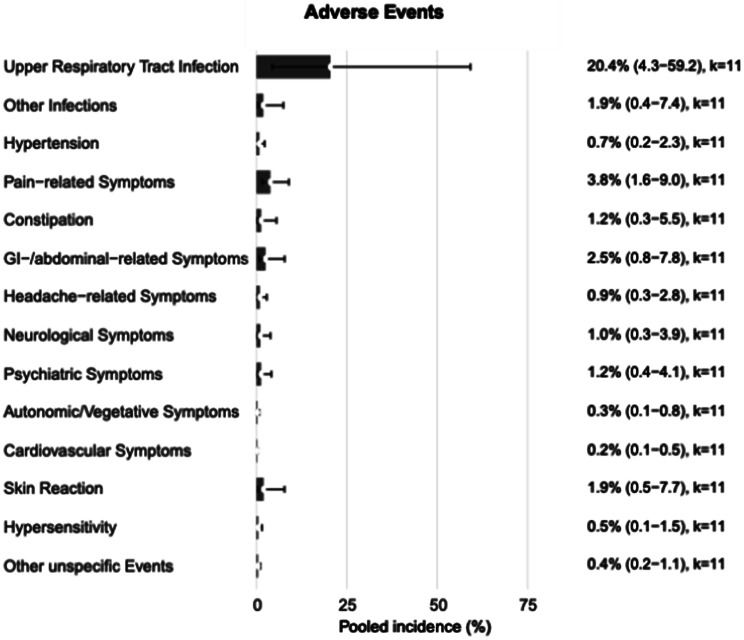
Incidence of reported adverse events. Pooled incidence (%) with 95% confidence intervals for each adverse event category, calculated using random-effects meta-analysis (REML τ^2^). Estimates are based on the latest or cumulative data for each study, with adverse event reporting thresholds retained as described in the original publications

The most frequently reported AE was upper respiratory tract infection (pooled incidence 20.4%, 95% CI 4.3–59.2; k = 11), followed by pain-related symptoms (3.8%, 1.6–9.0; k = 11) and GI/abdominal-related symptoms (2.5%, 0.8–7.8; k = 11) (Fig. 6). Most other categories, including constipation, headache-related symptoms, neurological symptoms, and psychiatric symptoms, had pooled incidences below 3%. Rarely reported categories (<2% pooled incidence) included autonomic/vegetative symptoms, cardiovascular symptoms, skin reactions, hypersensitivity, and other nonspecific events.

Of the eleven included studies, eight were conducted during the COVID-19 pandemic, although only two explicitly reported confirmed COVID-19 cases.

### Publication bias

Funnel plot inspection did not suggest substantial asymmetry for either total discontinuation or discontinuation due to AE (Supplementary Figures 2 and 3). Trim-and-fill analyses suggested the presence of potentially one missing study on the right side of the funnel plot of discontinuation due to AE, and led to a slightly altered pooled estimate compared with the original model. For total treatment discontinuation, Egger’s regression test indicated no evidence of small-study effects (*z* = 0.80, *p* = 0.42). For AE-related discontinuation, Egger’s test showed a nonsignificant trend towards asymmetry (*z* = −1.58, *p* = 0.11). Given the limited number of studies (k = 11) and the high heterogeneity (I^2^ > 90% for both outcomes), the power of these tests remains limited. Overall, evidence for publication bias was weak and did not materially alter the interpretation of the results.

## Discussion

This systematic review and meta-analysis synthesising eleven studies with an observation period of at least 12 months on anti-CGRP (receptor) mAbs found that overall drug discontinuation for any reason was moderate (23%), whereas discontinuation specifically due to AEs was consistently low (3%). Importantly, AE-related discontinuation remained stable throughout extended follow-up, suggesting durable tolerability of the anti-CGRP (receptor) mAbs in both clinical trial and real-world settings. No increase in AEs was seen over time.

Both open-label extension trials and real-world studies contributed to the overall evidence base, with the open-label extension trials including a larger number of participants. However, neither study type disproportionately influenced the pooled estimates. This contextualizes the findings across different study designs and enhances the interpretability of our results.

Our findings are broadly consistent with safety results from pivotal RCTs [24, 25] and open-label studies which reported AE-related discontinuation rates below 5% [26]. The present analysis extends this evidence by incorporating long-term follow-up and real-world data, encompassing over 4,000 patients across diverse clinical contexts. However, the data did not provide any direct comparison between the use of mAbs for less than and more than 12 months.

Though in this context, the consistently low AE-related discontinuation observed with anti-CGRP (receptor) mAbs stands in contrast to oral preventives such as propranolol, amitriptyline, and topiramate, which report discontinuation rates of 21–81% at 6 months and 45–93% at 12 months [27]. These discontinuation rates are not achieved even when patients are treated significantly longer with an anti-CGRP (receptor) mAbs.

Beyond AE-related discontinuation, a variety of additional factors contributed to treatment cessation, most commonly lack of efficacy and patient decision. Administrative factors such as reimbursement regulations were not reported but may also have influenced real-world discontinuation rates in several settings. The heterogeneous and incomplete reporting of these reasons across studies limits direct comparability and may contribute to variability in observed discontinuation patterns.

### Interpretation of time trends

Across studies, AE-related discontinuation appeared stable after the first year, with no clear signs of increasing safety concerns over time. Higher early discontinuation in some real-world cohorts likely reflects differences in patient selection and monitoring, after which rates plateaued. Although most patients reported at least one AE, the majority were non-serious and did not necessitate treatment cessation. However, these apparent trends should be interpreted cautiously. The analysis is primarily descriptive, based on heterogeneous reporting across studies with variable thresholds, timepoints, and definitions. As such, the findings are not robust enough to rule out late-emerging risks, but rather provide a broad overview suggesting stability in safety outcomes over prolonged exposure. 

### Adverse events

In our pooled incidence analysis, most AEs occurred at rates below 5%, except for upper respiratory tract infection, which was the most frequently reported event. A recent meta-analysis of randomised placebo-controlled trials found an increased relative risk of infection with CGRP-targeting therapies overall, particularly for galcanezumab and eptinezumab at higher doses [28]. Notably, upper respiratory tract infections were also commonly seen in placebo groups in anti-CGRP (receptor) mAb RCTs, very often with no difference to the comparator. Real-world data confirm these findings, with infection rates and types similar to those seen in clinical trials [29]. Nevertheless, the American Headache Society notes that infections are not considered a major safety concern in clinical practice, and discontinuation due to infection remains uncommon [30].

Importantly, most of the included studies were conducted during the COVID-19 pandemic, which may have increased the incidence of viral respiratory infections and affected study procedures such as follow-up visits and AE reporting, albeit in an identical way in parallel-group studies. As only two studies explicitly distinguished COVID-19 from other respiratory infections, the potential impact of the pandemic on reported AE rates cannot be fully evaluated and represents a limitation of the available evidence.

The incidence of constipation as a side effect is reported at approximately 10–20% for erenumab, about 3% for both eptinezumab and fremanzumab, and 3–10% for galcanezumab in clinical trials and real-world data [31, 32]. In our analysis, however, we observed a lower pooled incidence rate, even though most studies investigated erenumab. This discrepancy may reflect underreporting in extension and observational studies, differences in AE definitions and thresholds, or selective continuation of patients who tolerated treatment during earlier phases.

Pharmacovigilance data provide complementary insights into post-marketing safety [32, 33]. Reports were collected between launch and the third year of market availability. They indicate that constipation, injection site reactions, and alopecia are most common with erenumab. Injection site reactions occur more frequently with galcanezumab and fremanezumab, whereas eptinezumab is more frequently associated with fatigue and throat-related symptoms.

Compared to our pooled analysis, which showed AE are mild, non-serious events and have no high impact on discontinuation, pharmacovigilance data highlight additional signals such as alopecia and palpitations. These differences underscore the complementary nature of systematic study data and spontaneous reporting systems: while the former provide incidence estimates under controlled conditions, the latter are more sensitive to rare or unexpected events. Together, these approaches reinforce the overall tolerability of anti-CGRP (receptor) mAbs while emphasising the need for ongoing post-marketing surveillance to fully understand long-term safety.

### Limitations

This study has several limitations that should be considered when interpreting the findings.

First, data for most anti-CGRP mAbs, particularly fremanezumab, galcanezumab, and eptinezumab, were available from only one or two long-term studies, restricting the robustness and limiting the generalizability of pooled estimates. It also highlights the need for more clinical studies on long-term use.

Second, all included studies were judged to be at serious overall risk of bias, though the underlying reasons differed by design. Open-label extension trials only enrolled patients who tolerated and benefited from treatment during the randomised phase, leading to selective continuation of responders. This design inherently underestimates discontinuation and may obscure safety signals that would otherwise emerge in a broader population.

Third, AE reporting was highly heterogeneous. Some studies captured all AEs, while others reported only treatment-emergent AEs (TEAEs), and incidence thresholds varied considerably (e.g., > 5% of patients, ≥4 events per 100 patient-years, or no threshold). Furthermore, the time of onset of these AEs is not reported. Moreover, standardized definitions for specific AE categories were not consistently applied, which complicates the comparability of grouped outcomes and may have introduced misclassification. Phenotyping of patients by demographic characteristics (e.g., age, sex, race), AE-related parameters, and reasons for discontinuation is generally not provided. Consequently, no conclusions can be drawn on this aspect in the present review.

Despite these limitations, this review has notable strengths. The comprehensive literature search across multiple databases minimised the risk of missed studies, and the inclusion of both clinical trial and real-world evidence provides a broad perspective on long-term safety. The large cumulative sample size enhances the precision of pooled estimates, and the systematic grouping of AEs by clinical relevance allowed for a more coherent synthesis of diverse reporting practices.

## Conclusion

In summary, this systematic review and meta-analysis demonstrates that long-term treatment with anti-CGRP (receptor) mAbs is generally well tolerated, with AE-related discontinuation consistently low across agents and settings. The safety profile remained stable over extended follow-up periods, with most AE already reported in shorter observation periods of clinical trials and real-world studies and remained mild and non-serious. No new adverse event types were detected with longer treatment duration. These findings provide some evidence for the sustained good tolerability of anti-CGRP (receptor) mAbs in clinical practice, while underscoring the need for further clinical studies and continued real-world surveillance to detect rare outcomes and evaluate long-term safety.

## Electronic supplementary material

Below is the link to the electronic supplementary material.

Supplementary Material 1

## Data Availability

Data is provided within the manuscript or supplementary files.
