# Supplementary material for: Decoding the long-term safety of anti-CGRP (receptor) mAbs: a meta-analysis and systematic review
Source: J Headache Pain. 2026 Jan 3;27(1):10. doi: 10.1186/s10194-025-02256-0 (PMC12776973; doi:10.1186/s10194-025-02256-0)
Supplement: Supplementary file 1 — Supplementary Material 1 [file 10194_2025_2256_MOESM1_ESM.docx]

**Supplement Material**

**Decoding the Long-Term Safety of anti-CGRP (receptor) mAbs: A Meta-Analysis and systematic review**

**Supplementary Table 1: Incidence Threshold of Reported Adverse.** AE: Adverse Event; TEAE: Treatment-Emergent Adverse Event; OLEP: Open-Label-Extension-Phase; PTAP: , DBTP: Double-Blind-Treatment-Phase;

| **Author** | **Reported Adverse Event** |
| --- | --- |
| Yoshida et al. 2025 | AE |
| Göbel et al. 2024 | AE affecting over 5% |
| Gaul et al. 2024 | AEs by SOC at patient level |
| Reuter et al. 2024 | Summary of Treatment-Emergent Aes in the OLEP + PTAP |
| Ashina et al. 2023 | TEAEs in ≥1.5% of patient |
| Andreou et al. 2022 | Percentage and types of adverse events in patients treated with erenumab at month 6, 12, 18 and 24 |
| Ferrari et al. 2022 | Most frequently reported treatment-emergent AEs (per 100 patient-years) during the DBTP and OLEP, by preferred term |
| Sakai at al. 2021 | Frequent AEs ≥3.0/100 patient-years in the total erenumab group during OLTP. |
| Hirata et al. 2021 | Incidence of common treatment-emergent adverse events Common” was defined as incidence in the ALL treatment group ≥2% |
| Kudrow et al. 2021 | Study drug-related treatment-emergent adverse events over 2 years |
| Ashina et al. 2021 | Adverse Events with ≥5 patients per 100 patient-years in either erenumab 70 mg/140 mg group during the open-label treatment phase |
| Goadsby et al. 2021 | Proportion of Participants With Adverse Events and the Exposure-Adjusted Subject Rate per 100 Patient-Years in the DBTP and OLEP (Open-Label Analysis Set) |
| Tepper et al. 2020 | Events with ≥4.2 patients per 100 patient-years in the total erenumab group during open-label treatment phase; time at risk during the study is the time from first dose of erenumab through to onset of first event or the minimum (end-of-study date, last dose date + 112) |
| Ashina et al. 2019 | Most frequent AEs (>4.0/100 patient-years) |

**Supplementary Table 2: Reported Adverse Events and Assigned Categories.** SOC: system organ class. GI: Gastrointestinal.

| **Category** | **Reported adverse event** |
| --- | --- |
| **All Infections And Infestations** | Upper Respiratory Tract Infection |
|  | Other Infections |
|  | SOC Infection And Infestation |
| **Upper Respiratory Tract Infections** | Cough |
|  | Covid |
|  | Nasopharyngitis |
|  | Influenza |
|  | Pharyngitis |
|  | Bronchitis |
|  | Sinusitis |
|  | Upper Respiratory Tract Infection |
|  | Rhinitis |
|  | Viral Upper Respiratory Infection |
| **Other Infections** | Oral Herpes |
|  | Cystitis |
|  | Gastroenteritis |
|  | Urinary Tract Infection |
|  | Flu Like Symptoms |
| **Gastro-Intestinal-/Abdominal-Related Symptoms** | Nausea |
|  | Tooth Ache |
|  | Dental Caries |
|  | SOC GI |
|  | Dyspepsia |
|  | Abdominal Pain |
|  | Diarrhea |
|  | Abdominal Upper Pain |
| **Constipation** | Constipation |
| **Pain-Related Symptoms** | Oropharyngeal Pain |
|  | Ligament Sprain |
|  | Joint Stiffness And Pain |
|  | Body Aches |
|  | Arthralgia |
|  | Backpain |
| **Headache-Related Symptoms** | Typical Aura Without Headache |
|  | Headache |
|  | Migraine |
| **Neurological Symptoms** | Paresthesia |
|  | Tremor |
|  | SOC Musculoskeletal And Connective Tissue Disorders |
|  | Fatigue |
|  | SOC Nervous System Disorders |
|  | Vertigo |
|  | Dizziness |
| **Autonomic/Vegetative Symptoms** | Weight Gain |
|  | Weight Increase |
|  | Dry Mouth |
|  | Excessive Sweating |
| **Psychiatric Symptoms** | Depression |
|  | Lethargy |
|  | Mental Health Deterioration |
| **Hypertension** | Hypertension |
| **Cardiological Symptoms** | ECG Changes |
| **Skin Reaction** | Urticaria |
|  | Injection Site Reaction |
|  | Injection Site Pain |
|  | Erythema after Injection |
|  | Pruritus |
|  | Erythema |
|  | Eczema |
|  | SOC General Disorders and Administration Site Conditions |
| **Hypersensitivity** | Hypersensitivity |
|  | Anaphylactic Reaction |
|  | Flashes |
|  | Immunization Reaction |
| **Other Unspecific Events** | Post-Vaccination |
|  | Menopause |
|  | Conjunctivitis Allergic |

**Supplementary Table 3: Reasons for Treatment Discontinuation.**

*= reported as both patient and guard decision

| **Author (year)** | **Treatment Discontinuation** | **Insufficient efficacy** | **Patient decision** | **Physician decsion** | **New other migraine-treatment** | **Lost to follow-up** | **Pregnancy or active plans** | **Other** | **Adverse Event** |
| --- | --- | --- | --- | --- | --- | --- | --- | --- | --- |
| Reuter et al. 2024 | 72 | 30 | 26 * |  | 2 |  | 1 |  | 12 |
| Göbel et al. 2024 | 155 | 45 | 44 |  |  |  |  | 37 | 29 |
| Sakai at al. 2021 | 31 |  | 22 |  |  |  |  | 3 | 6 |
| Ashina et al. 2021 | 168 | 12 | 84 |  |  | 13 | 2 | 38 | 19 |
| Tepper et al. 2020 | 139 | 39 | 64 |  | 3 | 9 | 1 | 7 | 16 |
| Gaul et al. 2024 | 85 | 75 |  |  |  |  | 3 |  | 7 |
| Andreou et al. 2022 | 86 | 57 |  |  |  |  | 3 |  | 26 |
| Yoshida et al. 2025 | 15 | 6 | 7 |  |  |  | 1 |  | 1 |
| Hirata et al. 2021 | 33 |  | 10 | 1 |  |  | 1 | 2 | 19 |
| Kudrow et al. 2021 | 22 |  | 17 |  |  | 4 |  |  | 1 |
| Ashina et al. 2023 | 83 | 26 | 35 |  |  |  |  | 11 | 11 |

**Supplement Figure 1: Subgroup Analyses of AE-Related Discontinuation Rates by anti-CGRP (receptor) mAbs and Study Design.** Pooled incidence (%) with 95% confidence intervals for each adverse event category, calculated using random‐effects meta‐analysis (REML τ²). Estimates are based on the latest or cumulative data for each study, with adverse event reporting thresholds retained as described in the original publications.

**Supplement Figure 2: Funnel and trim-and-fill plots for total discontinuation.** Funnel plot illustrating the relationship between study precision and effect size for total treatment discontinuation across included trials (k = 11). Visual inspection revealed no substantial asymmetry. Egger’s regression test indicated no evidence of small-study effects (z = 0.80, p = 0.42).

**Supplement Figure 3: Funnel and trim-and-fill plots for adverse event (AE)-related treatment discontinuation.** Funnel plot illustrating the relationship between study precision and effect size for AE-related discontinuation across included trials (k = 11). Visual inspection suggested slight asymmetry on the right side of the plot, although Egger’s regression test did not indicate significant small-study effects (z = −1.58, p = 0.11). Trim-and-fill analysis identified one potentially missing study on the right side, resulting in a minimally altered pooled estimate compared with the original model. Overall, evidence for publication bias was weak and did not materially affect the interpretation of results**.**
